# Apoplastic histochemical features of plant root walls that may facilitate ion uptake and retention

**DOI:** 10.1515/biol-2021-0137

**Published:** 2021-12-31

**Authors:** Di Wu, Linbao Li, Chengdao Li, Bicheng Dun, Jun Zhang, Ten Li, Cunyu Zhou, Debao Tan, Chaodong Yang, Guiyun Huang, Xia Zhang

**Affiliations:** Rare Plants Research Institute of Yangtze River, China Three Gorges Corporation, Yichang, Hubei, 443000, China; Engineering Research Center of Ecology and Agriculture Use of Wetland, Ministry of Education, and Hubei Key Laboratory of Waterlogging Disaster and Agricultural Use of Wetland, Yangtze University, Jingzhou, Hubei 434025, China; Changjiang River Scientific Research Institute, Wuhan, Hubei 430010, China

**Keywords:** ions hyperaccumulator, histochemistry, oligotrophic environment, phytoremediation

## Abstract

We used brightfield and epifluorescence microscopy, as well as permeability tests, to investigate the apoplastic histochemical features of plant roots associated with ion hyperaccumulation, invasion, and tolerance of oligotrophic conditions. In hyperaccumulator species with a hypodermis (exodermis absent), ions penetrated the root apex, including the root cap. By contrast, in non-hyperaccumulator species possessing an exodermis, ions did not penetrate the root cap. *In vivo*, the lignified hypodermis blocked the entry of ions into the cortex, while root exodermis absorbed ions and restricted them to the cortex. The roots of the hyperaccumulators *Pteris vittata* and *Cardamine hupingshanensis*, as well as the aquatic invasives *Alternanthera philoxeroides*, *Eichhornia crassipes*, and *Pistia stratiotes,* contained lignin and pectins. These compounds may trap and store ions before hypodermis maturation, facilitating ion hyperaccumulation and retention in the apoplastic spaces of the roots. These apoplastic histochemical features were consistent with certain species-specific characters, including ion hyperaccumulation, invasive behaviors in aquatic environments, or tolerance of oligotrophic conditions. We suggest that apoplastic histochemical features of the root may act as invasion mechanisms, allowing these invasive aquatic plants to outcompete indigenous plants for ions.

## 1 Introduction

Several species in the genus *Pteris* (Pteridaceae), including *Pteris vittata*, hyperaccumulate ions such as arsenic (As) and chromium (Cr) [1,2,3,4]. The uptake, transport, translocation, and detoxification of heavy metals in the roots and fronds of these species have been well studied [2,3,4]. *Pteris* species have also evolved various anatomical features and hyperaccumulator functions to adapt to terrestrial, xeric, epiphytic, and rupicolous environments [3,4,5,6,7]. *Cardamine hupingshanensis* (Brassicaceae), which is found in Selenium (Se)-rich environments, is another well-known hyperaccumulator of ions, including Se and cadmium (Cd) [8,9,10]. Some invasive aquatic plants, including *Alternanthera philoxeroides* (Amaranthaceae), *Eichhornia crassipes* (Pontederiaceae), and *Pistia stratiotes* (Araceae), also hyperaccumulate ions: these species purify eutrophic water bodies and tend to outcompete indigenous plants in similar hostile environments [11,12,13,14,15,16,17,18,19,20]. Similarly, plants in the Proteaceae have evolved cluster roots with lignified or phenol-rich cortical walls to adapt to environments deficient in phosphorus and other nutrients [21,22,23]. Plants with cluster roots potentially facilitate alterations in plant community structure [24] and outcompete species without cluster roots [25]. Thus, cluster roots are highly desirable in crop breeding [26]. *Paspalum distichum* (Poaceae), a typical amphibious plant, is a non-hyperaccumulator with an endodermis and an exodermis in its roots [27].

In vascular plants, the lignified, suberized endodermis and exodermis act as apoplastic barriers, restricting water-solute exchange, reducing oxygen loss after submersion, and supporting adaptation to terrestrial environments [10,27,28,29,30,31,32,33,34,35,36,37]. The exodermis has Casparian bands in the primary walls and has suberin lamellae and/or lignin in the secondary walls [27,28,29,30,32,33,34,35,38]. Permeability tests showed that the lignified cortex and the hypodermis block ion exchange in *Alternanthera philoxeroides* and brassicas [18,39,40,41,42,43,44]. The cortical walls of the cluster roots in the Proteaceae contain soluble phenolic or lignin-like compounds that retain fluorescent agents (e.g., fluorol yellow 088); the presence of these compounds reflects an adaptation to nutrient deprivation [21,45,46,47,48,49,50,51,52]. In addition, the velamen, rhizodermis, and hairs of epiphytic orchids have pectins, which also facilitate ion uptake [53,54,55]. The surfaces of the mucilage hairs of *Brasenia schreberi* (Cabombaceae) have polysaccharides in various patterns that absorb berberine during different development stages *in vivo* [31,56].

In this study, we aimed to identify the apoplastic histochemical features of the root cortical walls that facilitate ion uptake and retention, leading to ion hyperaccumulation and reflecting an adaptation to nutrient-deprived environments. To identify these features, we investigated the roots of seven representative hyperaccumulator, invasive, and/or oligotrophic plants: the aerial species, *Pteris vittata* and *Chlorophytum comosum*; the wetland species, *Cardamine hupingshanensis* and *Paspalum distichum*; and the aquatic species, *Alternanthera philoxeroides, Eichhornia crassipes*, and *Pistia stratiotes*. We also tested the apoplastic permeability of *Pteris vittata* and *Paspalum distichum.* An improved understanding of these plant roots’ apoplastic histochemical features might help explain how these plants become invasive, tolerate oligotrophic conditions, and hyperaccumulate ions [4,5,8,10,11,12,19,20,22,23,25,26]. These data will support the development of plants that can be used for the phytoremediation of ion-contaminated soils and oligotrophic water. Our results will also provide suggestions for the breeding of crops that can outcompete weed species [3,8,11,12,14,19,23,25,26].

## 2 Materials and methods

### 2.1 Plant sourcing and collection

Mature specimens of *Pteris vittata*, *Paspalum distichum*, *Chlorophytum comosum*, *Cardamine hupingshanensis*, *Alternanthera philoxeroides*, *Eichhornia crassipes*, and *Pistia stratiotes* were identified in the Testing Ground of Yangtze University (Jingzhou City, Hubei Province, China) in October 2020. We collected samples of the adventitious aerial roots of *Pteris vittata*, which grow on walls in the cracks between bricks, and of *Chlorophytum comosum*, which propagate via shoots with adventitious aerial roots. We collected the roots of *Cardamine hupingshanensis* and *Paspalum distichum* from a wetland area. We collected the roots of *Alternanthera philoxeroides*, *Eichhornia crassipes*, and *Pistia stratiotes* from ponds. Ten roots were collected from each species of five plants and immediately fixed in formaldehyde-alcohol-acetic acid [57]. Eight fresh, intact specimens of *Pteris vittata* and *Paspalum distichum* were used for the apoplastic permeability tests [18,30,33,34,35].

### 2.2 Microstructure and histochemistry

Root tissues were sectioned freehand, using a two-sided razor blade, under a stereoscope (JNOEC JSZ6, China). Root sections were cut at 10 and 20 mm from the root tip, as well as at the point where the cortex began to slough off. Sections were divided into three sets, such that each set included sections of each plant and at same distance from the root tip. Each set of sections was then stained with one of three stains: 0.1% (w/v) berberine hemisulfate-aniline blue (BAB) to test for Casparian bands and lignin in the cell walls [38,58], phloroglucinol-HCl to test for lignin in the cell walls [59], and 0.02% (w/v) ruthenium red to test for pectin in the cell walls [55,60].

All sections were washed 2–3 times with sterile water, mounted with sterile water, and examined using brightfield microscopy under a Leica DME microscope (Germany). Specimens were photographed with a digital camera and a micrometer (Nikon E5400, Japan). Specimens stained with BAB were viewed under ultraviolet light on an Olympus IX71 epifluorescence microscope with excitation filter G 365 nm, absorption filter barriers U-WB (blue light), dichromatic mirror DM 500, compensation excitation filter BP 450–480, and compensation absorption filter BA 515. BAB-stained specimens were photographed using a digital camera and a micrometer (RZ200C-21, Ruizhi Cop., China) [27].

### 2.3 Apoplastic permeability

We tested the apoplastic permeability of whole fresh specimens of *Pteris vittata* and *Paspalum distichum*. We tested ion uptake using the apoplastic permeability tests of Seago et al., Meyer et al., and Meyer and Peterson [38,61,62], with modifications. In brief, we immersed the roots of the whole plants in the berberine solution without separating the roots from the plants; the plants remained intact. This modification allowed us to use the permeability tests to assess how the plants absorbed ions. Three intact plant roots were left unstained as the negative control. Three additional intact plants roots (tracer control) were immersed in 100 mL of 0.05% berberine hemisulfate for 1 h and washed with sterile water. The final three intact plant roots were immersed in 100 mL of 0.05% berberine hemisulfate for 1 h, washed with sterile water, immersed in 0.05 M potassium thiocyanate for 0.5 h, and washed again with sterile water. Roots were sectioned freehand and viewed under UV light as described by Seago et al. [38].

## 3 Results and discussion

**Figure 1:**
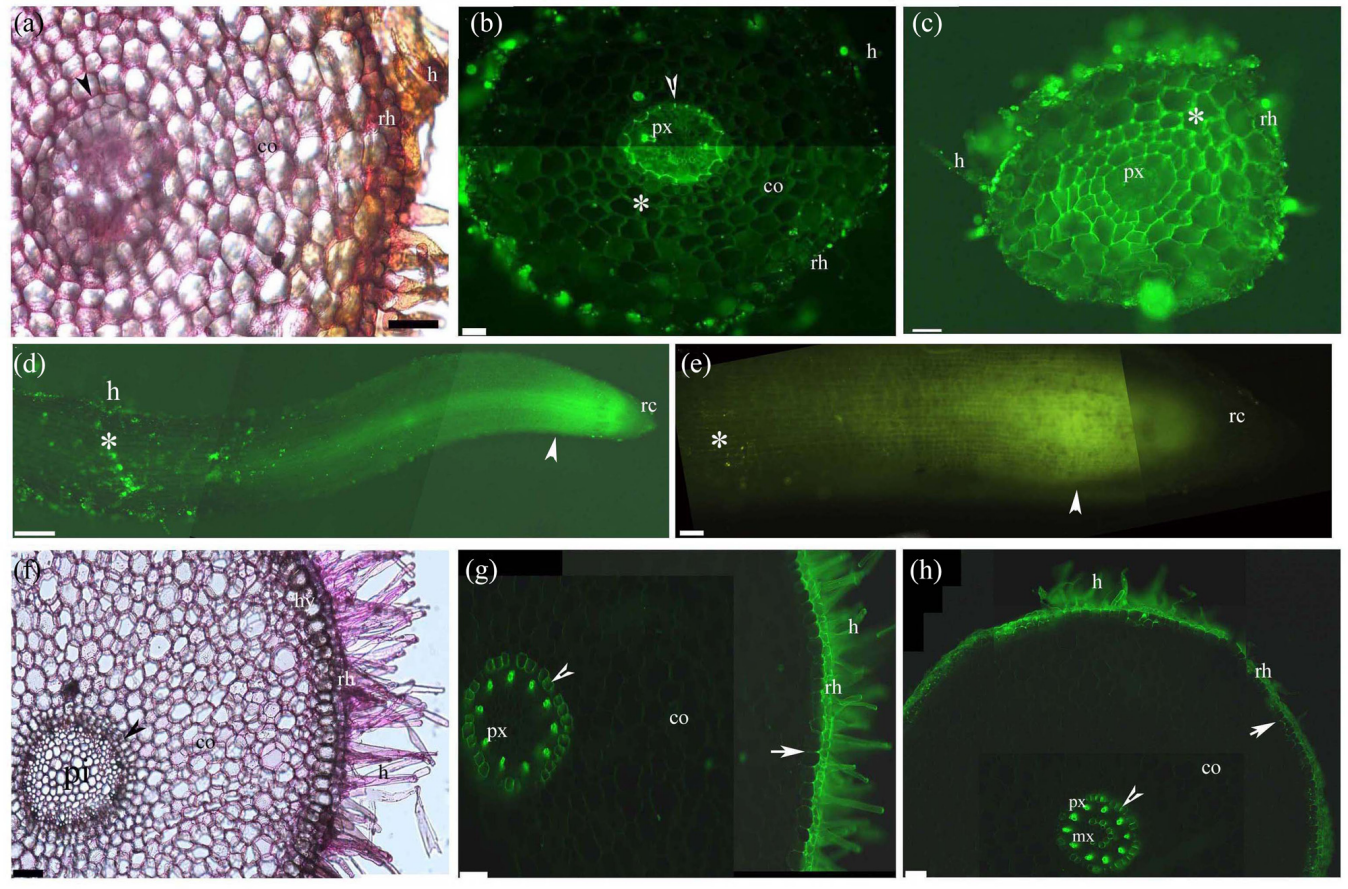
Photomicrographs of the adventitious roots of (a–d) *Pteris vittata,* (e) *Paspalum distichum*, and (f–h) *Chlorophytum comosum*. Scale bars = 50 μm. (a) Sectioned at 10 mm from root tip. Endodermis (arrowhead), cortex, rhizodermis, and hairs. Stain: ruthenium red. (b) Sectioned at 10 mm from root tip. Protoxylem, endodermis (arrowhead), lignified cortex (*), cortex, rhizodermis, and hairs. Stain: BAB. (c) Sectioned at 10 mm from root tip. Protoxylem, lignified thickened cortex (*), hypodermis, rhizodermis, and hairs, showing heavy accumulation of berberine thiocyanate. Stain: berberine (apoplastic tracer) and potassium thiocyanate. (d) Root tip showing root cap and entrance of berberine thiocyanate (arrowhead) close to the root tip; rhizodermis and hairs showing berberine thiocyanate accumulation (*). Stain: berberine (apoplastic tracer) and potassium thiocyanate. (e) Root tip showing root cap and entrance of berberine thiocyanate (arrowhead) close to the root tip; rhizodermis with limited berberine thiocyanate accumulation (*). Stain: berberine (apoplastic tracer) and potassium thiocyanate. (f) Sectioned at 10 mm from root tip. Pith, endodermis (arrowhead), cortex, hypodermis, rhizodermis, and hairs. Stain: ruthenium red. (g) Sectioned at 10 mm from root tip. Protoxylem, endodermis (arrowhead), cortex, exodermis (arrow), rhizodermis, and hairs. Stain: BAB. (h) Sectioned at 20 mm from root tip. Protoxylem, metaxylem, endodermis (arrowhead), cortex, exodermis (arrow), rhizodermis, and hairs. Stain: BAB. Abbreviations: ae, aerenchyma; co, cortex; h, hairs; hy, hypodermis; ic, intercellular space; mx, metaxylem; pa, parenchyma; pi, pith; px, protoxylem; rc, root cap; rh, rhizodermis; sc, sclerenchyma layer; sx, secondary xylem.

At 10 mm from the tips of the adventitious aerial roots of *Pteris vittata,* the root wall contained pectins from the endodermis to the rhizodermis and hairs (Figure 1a); the inner cortex had lignin-rich sclerenchyma layers and retained berberine around the endodermis (Figure 1b); and the surfaces of the rhizodermis and hairs accumulated substantial amounts of berberine or berberine thiocyanate crystals (Figure 1b–d). Berberine penetrated to the cortex of the *Pteris vittata* roots close to the root tips (Figure 1c and d), as indicated by the intense yellow fluorescence from the rhizodermis to the cortex. Similarly, intense yellow fluorescence was observed close to the tips of the roots of *Paspalum distichum* (Figure 1e), but berberine did not penetrate the root cap of this species. The walls of the adventitious aerial roots of *Chlorophytum comosum* also contained pectins from the endodermis to the rhizodermis and hairs (Figure 1f). Similar to *Pteris vittata*, the surfaces of the rhizodermis and hairs accumulated large amounts of berberine before metaxylem development (Figure 1g). After metaxylem development, the hairs were nearly sloughed off, but the exodermis and the rhizodermis surface continued to retain berberine (Figure 1h).

**Figure 2:**
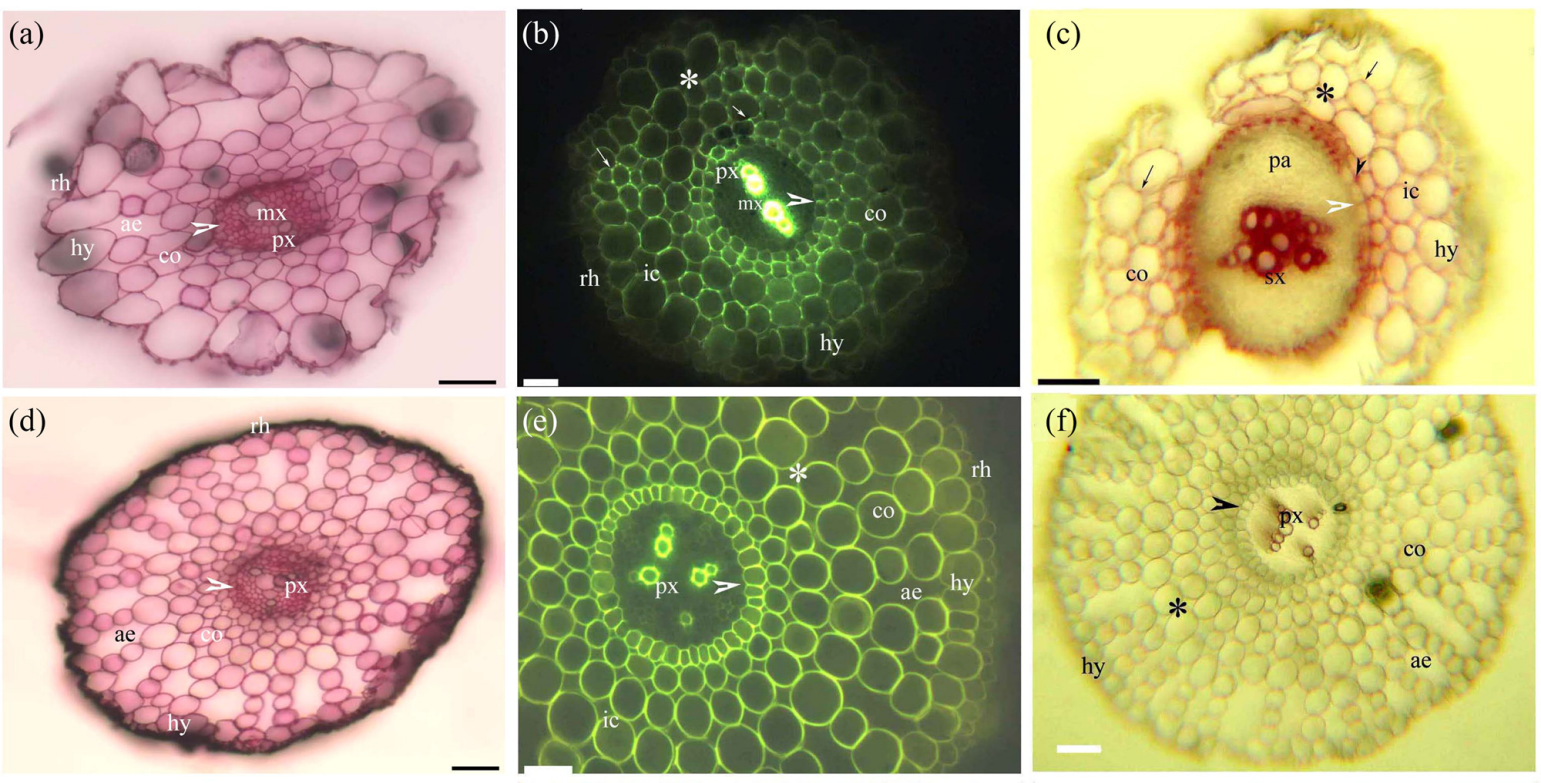
Photomicrographs of the adventitious roots of (a–c) *Cardamine hupingshanensis* and (d–f) *Alternanthera philoxeroides*. Scale bars = 50 μm. (a) Sectioned at 10 mm from root tip. Protoxylem, metaxylem, endodermis (arrowhead), cortex, aerenchyma, hypodermis, and rhizodermis. Stain: ruthenium red. (b) Sectioned at 10 mm from root tip. Protoxylem, metaxylem, endodermis (arrowhead), cortex, lignified cortex (*), cortical lignified Φ thickenings (arrows), intercellular space, hypodermis, and rhizodermis. Stain: BAB. Image from [10] used with the permission of *Open Life Sciences*. (c) Sectioned at 50 mm from root tip. Secondary xylem, parenchyma, endodermis (white arrowhead), cortex, inner cortical lignified Φ thickening (black arrowhead), outer cortical lignified Φ thickenings (black arrows), lignified cortex (*), intercellular space, and hypodermis. Stain: phloroglucinol-HCl. Image from [10] used with the permission of *Open Life Sciences*. (d) Sectioned at 10 mm from root tip. Protoxylem, endodermis (arrowhead), cortex, aerenchyma, hypodermis, and rhizodermis. Stain: ruthenium red. (e) Sectioned at 10 mm from root tip. Protoxylem, endodermis (arrowhead), cortex, aerenchyma, lignified cortex (*), hypodermis, and rhizodermis. Stain: BAB. Image from [18] used with the permission of *Flora*. (f) Sectioned at 10 mm from root tip. Protoxylem, endodermis (arrowhead), cortex, aerenchyma, lignified cortex (*), and hypodermis. Stain: phloroglucinol-HCl.

**Figure 3:**
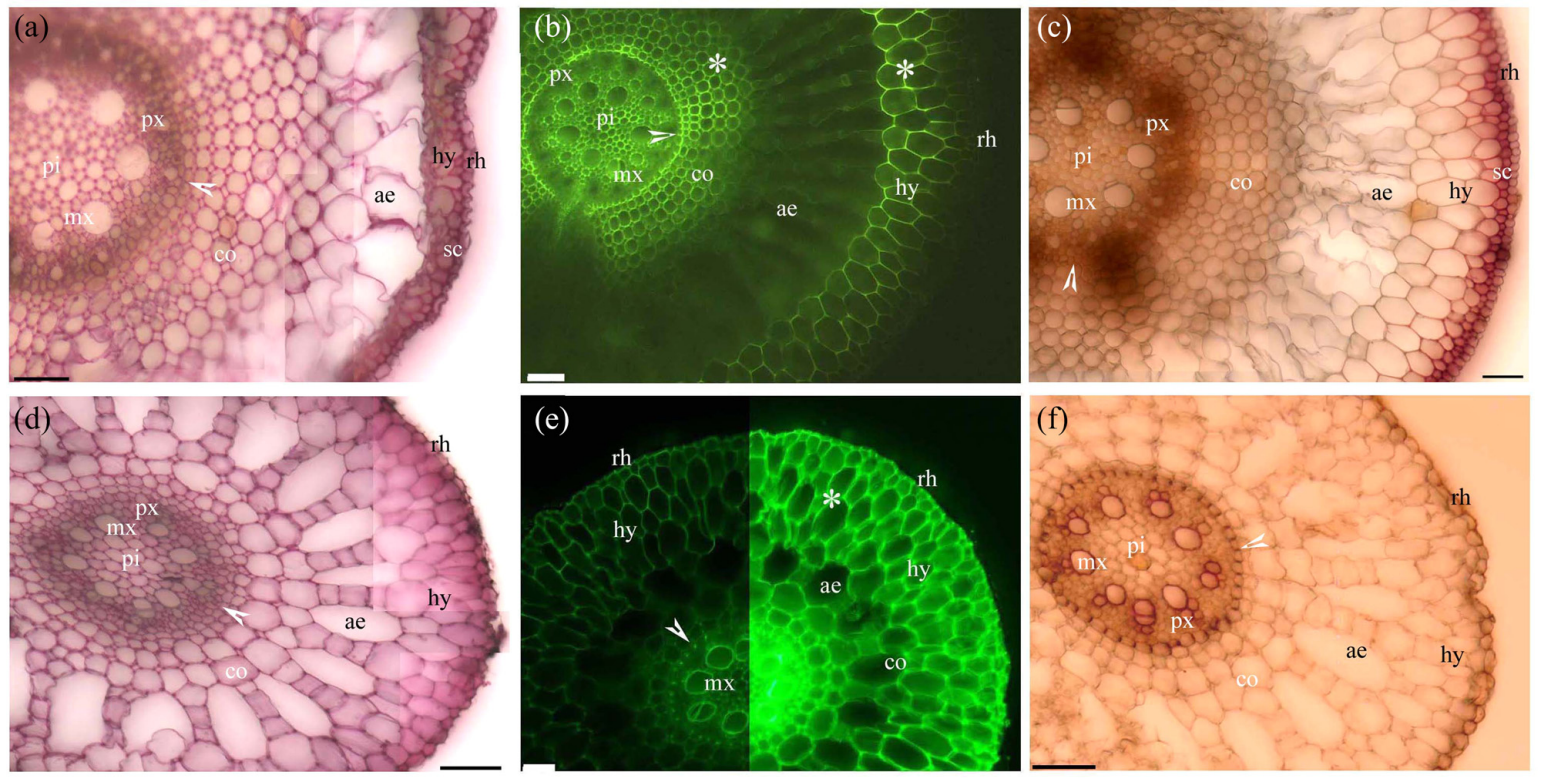
Photomicrographs of the adventitious roots of (a–c) *Eichhornia crassipes* and (d–f) *Pistia stratiotes*. Scale bars = 50 μm. (a) Sectioned at 10 mm from root tip. Pith, protoxylem, metaxylem, endodermis (arrowhead), cortex, aerenchyma, hypodermis, sclerenchyma layer, and rhizodermis. Stain: ruthenium red. (b) Sectioned at 10 mm from root tip. Pith, protoxylem, metaxylem, endodermis (arrowhead), cortex, aerenchyma, lignified cortex (*), hypodermis, and rhizodermis. Stain: BAB. (c) Sectioned at 10 mm from root tip. Pith, protoxylem, metaxylem, endodermis (white arrowhead), cortex, aerenchyma, lignified cortex (*), hypodermis, sclerenchyma layer, and rhizodermis. Stain: phloroglucinol-HCl. (d) Sectioned at 10 mm from root tip. Pith, protoxylem, metaxylem, endodermis (arrowhead), cortex, aerenchyma, hypodermis, and rhizodermis. Stain: ruthenium red. (e) Sectioned at 10 mm from root tip. Metaxylem, endodermis (arrowhead), cortex, aerenchyma, lignified cortex (*), hypodermis, and rhizodermis. Stain: BAB. (f) Sectioned at 10 mm from root tip. Pith, protoxylem, metaxylem, endodermis (arrowhead), cortex, aerenchyma, lignified cortex (*), hypodermis, and rhizodermis. Stain: phloroglucinol-HCl.

Before the cortex sloughed off, the adventitious roots of *Cardamine hupingshanensis* had pectins and lignin with even and Φ thickenings from the endodermis to the rhizodermis walls (Figure 2a–c). Similarly, pectins and lignified even thickenings were found from the endodermis to the rhizodermis walls in the adventitious roots of the aquatic plants *Alternanthera philoxeroides* (Figure 2d–f), *Eichhornia crassipes* (Figure 3a–c), and *Pistia stratiotes* (Figure 3d–f). In the adventitious roots of *Alternanthera philoxeroides* (Figure 2d–f) and *Pistia stratiotes* (Figure 3d–f), the cortex had typical radial schizogenous aerenchyma, while in the adventitious roots of *Cardamine hupingshanensis* (Figure 2a–c) and *Eichhornia crassipes* (Figure 3a–c), the cortex had radial lysigenous aerenchyma. The hypodermis of *Eichhornia crassipes* had lignified sclerenchyma layers (Figure 3a and c).

The endodermis and the exodermis are key impermeable apoplastic barriers that are common in vascular plant roots [28,29,36,37,63,64,65,66,67,68]. Apoplastic barriers can be histochemically characterized by the presence of Casparian bands, suberin lamellae, and lignin; these barriers protect tissues from oxygen shortages and inhibit water-solute exchanges [18,23,30,34,35,69,70,71,72,73,74]. All the species examined in this study had roots with an endodermis, but only the roots of *Paspalum distichum* and *Chlorophytum comosum* had an exodermis [27,75]. The roots of the other species included in this study (*Pteris vittata*, *Cardamine hupingshanensis*, *Alternanthera philoxeroides*, *Eichhornia crassipes*, and *Pistia stratiotes*) lacked an exodermis but possessed a hypodermis with lignin, as has been described in a variety of other plants, including *Adiantum reniforme* var. *sinense*, *Brassica* sp., *Oenanthe javanica*, *Lycopodium obscurum*, *Pelargonium hortorum*, *Platycerium bifurcatum,* and *Selaginella* sp. [10,37,39,40,41,42,43,44,66,76,77,78,79]. The cluster roots of various genera in the Proteaceae (e.g., *Banksia, Grevillea,* and *Hakea*) have a hypodermis containing soluble phenolic or lignin-like compounds, which have similar histochemical features with lignin of hypodermis in this studied species [21,49,50,51,52].

Lignified Φ and even thickenings that are distributed between the endodermis and the hypodermis of the cortical walls are typical of roots in the Brassicaceae, including in the genera *Brassica*, *Noccaea*, and *Cardamine* [10,39,40,41,42,43,44]. These thickenings act as barriers to ion transport. Unlike plants in the Brassicaceae, *Pelargonium hortorum* has larger Φ thickenings at the hypodermis [77]. The roots of *Platycerium bifurcatum*, *Pleopeltis* sp., and *Doryopteris triphylla* have lignified sclerenchyma layers around the endodermis [78,79,82,83,84], while the roots of *Selaginella* sp. have an exodermis [79]. In the heavy metal hyperaccumulator *Noccaea caerulescens,* the inner cortical walls of roots contain pectins and lignin [43]. Here lignified cortical thickenings were found in the roots of *Pteris vittata* [85], *Cardamine hupingshanensis* [10], *Alternanthera philoxeroides* [18], *Eichhornia crassipes*, and *Pistia stratiotes*.

In *Pteris vittata* and *Chlorophytum comosum,* the rhizodermis and hair walls contained pectins and accumulated a large amount of berberine. Similarly, the orchid root velamen also contains pectins and accumulates ions [53,54,55]. The root rhizodermis and hair walls of the hyperaccumulating ecotype of *Sedum alfredii* accumulated substantial Leadmium Green AM dye [80]; roots in this species also have thin inner cortical walls that contain large amounts of highly methylated pectin [81]. The root surfaces of *Chlorophytum comosum* retained berberine, similar to retention of polysaccharides by the smooth, immature mucilage hairs of *Brasenia schreberi*; the retention of berberine thiocyanate grains by the *Pteris vittata* root surfaces was similar to that of the mature mucilage hairs of *Brasenia schreberi in vivo* [31,56]. The rhizodermis surface retains little berberine in *Adiantum reniforme* var. *sinense* [37] and retains none in *Metasequoia glyptostroboides* [32], *Cardamine hupingshanensis* [10], and *Alternanthera philoxeroides* [18]. By contrast, our results showed that the surface of the root rhizodermis in *Pteris vittata* and *Chlorophytum comosum* retained substantial berberine. Pectins were present from the endodermis to the rhizodermis walls in *Cardamine hupingshanensis*, *Alternanthera philoxeroides*, *Eichhornia crassipes*, and *Pistia stratiotes*. However, pectins are only found in the peri-endodermal thickenings of *Noccaea caerulescens* [39,43].

In the apoplastic permeability test, the berberine tracer penetrated to the cortex of both *Pteris vittata* (exodermis absent) and *Paspalum distichum* (exodermis present) near the root tips [27,75], similar to what has been shown in *Iris germanica* (exodermis present) [61,86]. The berberine tracer also penetrated the root caps of *Pteris vittata,* similar to the results in *Vicia faba* (exodermis absent) [86]. However, the berberine tracer was unable to penetrate the root cap of *Paspalum distichum*, similar to what has been shown in *Zea mays* (exodermis present) and *Iris germanica* (exodermis present) [61,86]. Many berberine thiocyanate grains adhered to the mature hypodermis of *Pteris vittata*. By contrast, few berberine thiocyanate grains adhered to the mature exodermis of *Paspalum distichum* at the root surface [27,75]. The lignified hypodermis of *Alternanthera philoxeroides* blocks the entrance of ions into the cortex [18]. The root exodermis has only been shown to absorb berberine *in vivo* in *Phalaris arundinacea*, *Zizania latifolia,* and *Artemisia* spp. [30,34,35].

Based on the apoplastic histochemical features of the roots and their permeability, we hypothesize that the root hairs of *Pteris vittata* and *Chlorophytum comosum* have pectins that capture ions from the atmosphere, which helps these plants to survive in an oligotrophic aerial environment. Like *Pteris vittata* and *Chlorophytum comosum,* the epiphytic Orchidaceae use pectins to capture ions from the atmosphere [53,54,55]. Similarly, *Brasenia schreberi* uses polysaccharides to capture ions [7,31,39,53,54,55,56]. The carpet-like root system of *Pteris vittata* has many adventitious roots that absorb captured ions, leading to the hyperaccumulation of ions such as As and Cr [1,2,3,4,7,31,39,53,54,55,56,80,81,86,87]. We suggest that the lignified thickenings and pectins in the roots of *Cardamine hupingshanensis*, *Alternanthera philoxeroides*, *Eichhornia crassipes*, and *Pistia stratiotes* may trap ions before the hypodermis matures. These ions are then retained in the lignified walls, giving these species a competitive advantage over indigenous plants, particularly in oligotrophic environments [8,9,10,11,12,13,14,15,16,17,18,19,20,21,22,23,24,25,26,39,40,41,42,43,44]. Finally, the dense, fine roots of *Cardamine hupingshanensis* may allow the plant to hyperaccumulate Se in a manner that is similar to the hyperaccumulation of Cd in the dense root hairs of certain *Arabidopsis thaliana* genotypes [10,88] and in the phenol-rich cluster roots of species in the Proteaceae [21,22,23,24,25,49,50,51,52]. This ability to hyperaccumulate ions supports the adaptation of these plants to phosphorus deprivation and/or nutrient-poor environments [22,23,26,50,52].

## 4 Conclusion

Histochemical analyses indicate that pectins and lignin are present in several parts of the plant root walls, including the cortex, endodermis, exodermis or hypodermis, rhizodermis, and hairs [8,9,10,11,12,13,14,15,16,17,18,19,20,31,39,40,41,42,43,44,52,53,54,55,56,80,81,86]. These compounds, including the polysaccharides and phenolics, may facilitate ion uptake and retention in plants [8,9,10,11,12,13,14,15,16,17,18,19,20,21,22,23,24,25,26,27,28,29,30,31,34,35,39,40,41,42,43,44,52,53,54,55,56, 61,66,75,80,81,86]. In hyperaccumulator species without an exodermis (hypodermis), ions penetrate the root apex as well as the root cap [66,86]. By contrast, ions do not penetrate the root cap in non-hyperaccumulator species possessing an exodermis [27,61,66,75,86]. It has been shown *in vivo* that the lignified hypodermis of the root blocks the entry of ions into the cortex [18,86], while the root exodermis absorbs ions, trapping them within the exodermis walls [30,34,35,61,75,86]. The root hairs of *Pteris vittata* and *Chlorophytum comosum* are pectin-rich, reflecting an adaptation to the oligotrophic aerial environment [31,39,43,53,54,55,56,80,81]. The roots of the hyperaccumulators *Pteris vittata* and *Cardamine hupingshanensis*, as well as those of the invasive aquatic plants *Alternanthera philoxeroides*, *Eichhornia crassipes*, and *Pistia stratiotes,* have lignin and pectins in the cortex and rhizodermis; these structures may trap and store ions before hypodermis maturation. This hyperaccumulation of ions supports the survival of these plants in oligotrophic environments [8,9,10,11,12,13,14,15,16,17,18,19,20,21,22,23,24,25,26,39,40,41,42,43,44,52]. We suggest that the apoplastic histochemical features of invasive aquatic plant roots may allow such plants to acquire ions more efficiently than indigenous plants, and these features can thus be considered invasive mechanisms [11,12,13,14,15,16,17,18,19,20]. The histochemical features associated with hyperaccumulation are highly desirable for crop improvement, as well as when designing plants for the phytoremediation of ion-contaminated soils and for the population of eutrophic environments [11,12,13,14,15,16,17,18,19,20,26,50,51,52,89,90,91,92,93,94].
